# Evaluation of the RNA Silencing Suppression Activity of Three Cherry Virus F-Encoded Proteins

**DOI:** 10.3390/plants13020264

**Published:** 2024-01-17

**Authors:** Leonidas Lotos, Asimina Katsiani, Nikolaos I. Katis, Varvara I. Maliogka

**Affiliations:** Plant Pathology Laboratory, School of Agriculture, Faculty of Agriculture, Forestry and Natural Environment, Aristotle University of Thessaloniki, 54124 Thessaloniki, Greece; llotos@agro.auth.gr (L.L.); meniakats@yahoo.gr (A.K.); katis@agro.auth.gr (N.I.K.)

**Keywords:** RNA silencing, suppressor proteins, CVF, fabavirus

## Abstract

Cherry virus F (CVF) is a newly emerged sweet cherry virus. CVF has been identified in a small number of countries and it has not been associated with discrete symptomatology. RNA silencing is a natural defense mechanism of plants against invaders that degrades viral RNA in a sequence-specific manner. As a counter-defense, plant viruses encode one or more RNA silencing suppressors (RSSs) interfering with the silencing pathway via several mechanisms. To identify putative RSSs, the three proteins (MP, CPL, CPS) encoded by the RNA2 of CVF were selected and separately cloned into the binary vector pART27. The clones were used for transient expression experiments in *Nicotiana benthamiana* leaves, using co-agroinfiltration with a GFP-expressing vector. In both CPL and CPS, a rapid decrease in fluorescence was recorded, comparable to the negative control, whereas the MP of CVF retained the GFP’s fluorescence for a few days longer even though this was observed in a small number of infiltrated leaves. Further experiments have shown that the protein was not able to inhibit the cell-to-cell spread of the silencing signal; however, a putative interference with systemic silencing was recorded especially when the induction was carried out with double-stranded GFP RNA. Overall, our results indicate that the MP of CVF is putatively implicated in the suppression of RNA silencing, though further experimentation is needed to unveil the exact mode of action.

## 1. Introduction

RNA silencing is a sequence-specific RNA-mediated regulatory process that has a major antiviral role in plants. Silencing is induced locally and a mobile signal moves from cell to cell through plasmodesmata and across long distances to trigger silencing systemically [1]. As a counter-defense, plant viruses encode proteins acting as RNA silencing suppressors (RSSs) that interfere with the silencing pathway via diverse mechanisms at different temporal stages [2]. RSSs have been identified in most plant virus genera and are structurally diverse and required for virus accumulation, spread, and virulence in infected plants [3]. 

Cherry virus F (CVF) (tentative species in family *Secoviridae*, genus *Fabavirus*) is a recently identified sweet cherry (*Prunus avium*) and Japanese plum (*Prunus mume*) infecting virus reported in Europe and Korea [4,5]. CVF was also found in bee bread collected from bee hives in British Columbia (Canada), which implies the presence of the virus in that region as well [6]. It has a bipartite genome with each RNA coding for a polyprotein which, after translation, is proteolytically processed into functional proteins. Studies on other fabaviruses and comoviruses have indicated that the three proteins (movement protein—MP, large coat protein—CPL, small coat protein—CPS) produced from RNA2 could act as possible RNA silencing suppressors (RSSs) [7]. 

Identification of RNA silencing suppressors is based on their transient expression after delivery by *Agrobacterium tumefaciens* (agroinfiltration) in *Nicotiana benthamiana* plants (wild type, WT) and the ability of the protein under study to preserve the level of green fluorescent protein (GFP) expression under silencing conditions. Transgenic plants expressing GFP (16c line) are also used for the transient expression of putative suppressors. As they provide a rapid, versatile, and convenient way for achieving a very high level of gene expression in leaves, agrobacterium-mediated transient expression systems were selected to be used for the identification of putative suppressors encoded by the genomes of CVF. This approach was also used to study interference with the movement of silencing signal in the plant. Overall, our results indicate a potential role of the MP of CVF in the suppression of the RNA silencing pathway.

## 2. Results

### 2.1. Expression Clones

CVF PCR products were ligated to pART7[EcoRI-BamHI,P-] (pART7 digested with EcoRI at the 5′ end and BamHI at the 3′ end and dephosphorylated) in order to produce p7:35S:CVF-MP, p7:35S:CVF-CPL, p7:35S:CVF-CPS. The p7 plasmids were digested with NotI and the fragments were ligated to pART27[NotI,P-] to produce p27:35S:CVF-MP, p27:35S:CVF-CPL, and p27:35S:CVF-CPS. p27 constructs were transformed into C58C1 competent cells and the final clones of At:35S:CVF-MP, At:35S:CVF-CPL, and At:35S:CVF-CPS were obtained.

### 2.2. Evaluation of the Local RNA Silencing Suppression Capacity of the Three CVF Proteins 

**Figure 1:**
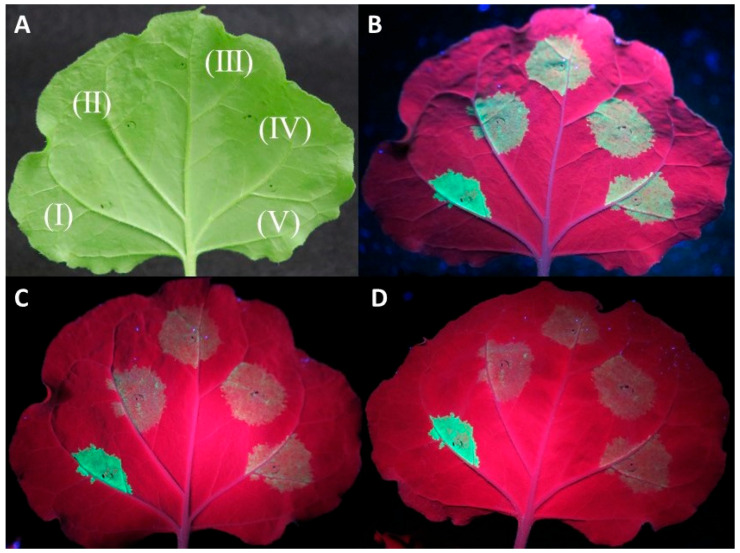
Investigation of the capacity of CVF MP, CPL, and CPS proteins to suppress RNA silencing using an *Agrobacterium tumefaciens* co-infiltration assay. Agroinfiltrations of WT *Nicotiana benthamiana* leaves with At:35S:p19/At:35S:GFP (I), At:35S:pART27/At:35S:GFP (II), At:35S:CVF-CPL/At:35S:GFP (III), At:35S:CVF-CPS/At:35S:GFP (IV), and At:35S:CVF-MP/At:35S:GFP (V). (**A**) leaf under white light, (**B**–**D**) the same leaf under UV light at 3 days post infiltration (dpi) (**B**), 4 dpi (**C**), and 5 dpi (**D**) exhibiting GFP fluorescence in the infiltrated spots.

Co-agroinfiltration assays of the protein expression clones with a GFP-expressing plasmid in WT *N. benthamiana* plants showed in most replicates a rapid drop in GFP fluorescence for the three tested proteins similar to the negative control (empty pART27 vector) at 4–5 days post infiltration (dpi) (Figure 1). 

**Figure 2:**
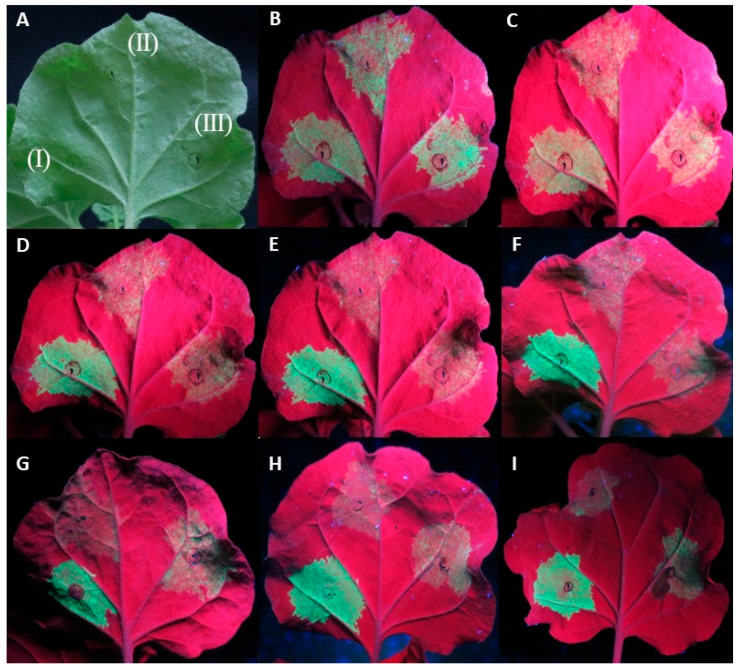
Agroinfiltrations of WT *Nicotiana benthamiana* leaves with At:35S:p19/At:35S:GFP (I), At:35S:pART27/At:35S:GFP (II), and At:35S:CVF-MP/At:35S:GFP (III). (**A**) leaf under white light, (**B**–**F**) same leaf under UV light at 4 days post infiltration (dpi) (**B**), 5 dpi (**C**), 6 dpi (**D**), 7 dpi (**E**), and 8 dpi (**F**). Leaves of different replicates exhibiting variable levels of fluorescence at 6 dpi for CVF-MP (**G**–**I**).

In a small number of replicates (11/92) of the At:35S:CVF-MP, a slight lag in the drop of fluorescence was observed; however, neither the intensity of the fluorescence nor the number of days that the florescence remained present were consistent on the observed leaves (Figure 2). 

### 2.3. Relative Expression of GFP

**Figure 3:**
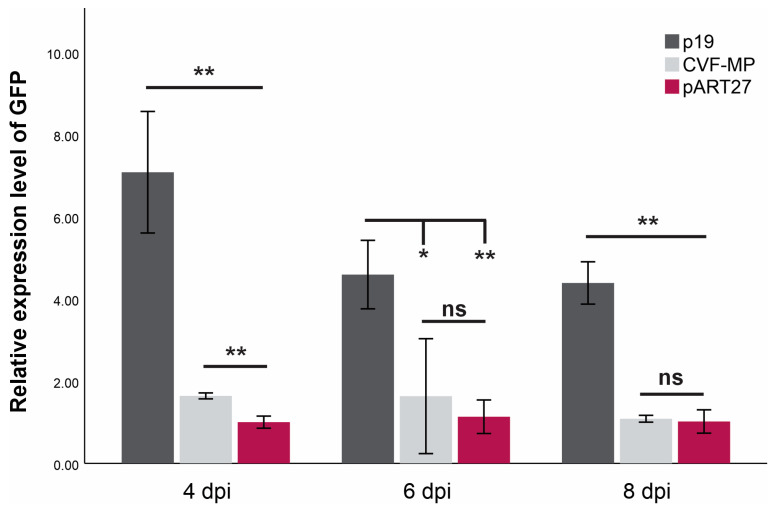
Mean relative expression levels of GFP mRNA from three biological replicates (wild type *Nicotiana benthamiana* leaves) at 4, 6, and 8 days post infiltration (dpi) using L23 as an internal control for normalization. Significance was tested using an independent *t*-test with significance *p* < 0.01 (**) or <0.05 (*), ns = non-significant. Whiskers indicate SD.

For all treatments, the onset of GFP fluorescence was observed on the third day post infiltration, with the empty vector control (pART27) levels dropping rapidly after the 4th day and the P19 controls retaining the fluorescence until the last day of observation (11 dpi). The CVF-MP treatment exhibited variable results, as fluoresence intensity for the majority of infiltrated spots was greatly reduced on the 4th day with complete loss after the 6th day, but a couple of spots retained high fluorescence up to 6 dpi. At all three timepoints, the expression levels of P19 were significantly higher than CVF-MP (Figure 3) with their relative expression means (±SD) being 7.08 ± 1.48, 4.59 ± 0.83, and 4.39 ± 0.51 for 4, 6, and 8 dpi, respectively. CVF-MP levels, 1.64 ± 0.07 (mean ± SD), 1.6 ± 1.39, and 1.08 ± 0.08 for 4, 6, and 8 dpi, respectively, differed significantly from the pART27 control only on 4 dpi (Figure 3).

### 2.4. Interference with Local Cell-to-Cell Movement and Systemic RNA Silencing

**Figure 4:**
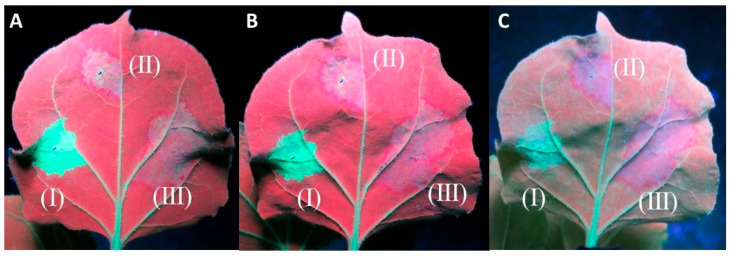
Agroinfiltrations of 16c *Nicotiana benthamiana* leaves with At:35S:p19/At:35S:GFP (I), At:35S:pART27/At:35S:GFP (II), and At:35S:CVF-MP/At:35S:GFP (III) for the evaluation of cell-to-cell inhibition of silencing signal. The photos were taken at 8 days post infiltration (dpi) (**A**), 9 dpi (**B**), and 13 dpi (**C**). Both the empty vector and CVF-MP infiltration spots formed a red halo on the periphery of the infiltration spot, associated with RNA silencing.

In the experiments evaluating the cell-to-cell movement of the silencing signal, all twenty of the 16c plants infiltrated displayed a red halo around the infiltration spots of At:35S:CVF-MP and At:35S:pART27 (empty vector), indicating that CVF-MP cannot stop the movement of the silencing signal in the adjacent cells (Figure 4). In the experiments evaluating the systemic silencing of GFP, when GFP silencing was induced with single stranded (ss) GFP RNA using At:35S:GFP, 7 out of 18 16c plants co-infiltrated with the construct expressing CVF-MP did not show any systemic silencing in the newly developed tissues at 40 dpi (Supplementary Figure S1). However, when the silencing was induced with At:35S:GFP-hp, the newly developed leaves exhibited green fluorescence up to 40 days post infiltration in the majority of CVF-MP co-infiltrated plants (13/19) (Supplementary Figure S2). In all cases, the 16c control plants infiltrated with At:35S:pART27/At:35S:GFP or At:35S:pART27/At:35S:GFP-hp exhibited the anticipated systemic silencing, whereas the plants infiltrated with At:35S:p19/At:35S:GFP or At:35S:p19/At:35S:GFP-hp retained GFP fluorescence on the upper leaves until the last day of observations (40 dpi).

### 2.5. In Silico Analysis of the Protein Genes

The in silico analysis showed that the proteins tested do not possess any WG/GW motifs; therefore, it is rather impossible to bind Ago proteins.

To study the diversity of CVF MP, sequence alignments were performed between the MP proteins of several available virus isolates. For this purpose, 10 complete amino acid sequences, including isolate SwC-G15-3, were retrieved from GenBank and used. Comparative analysis revealed 91 to 95% amino acid sequence identity between the isolates (Supplementary Figure S3) and highlighted that the central part of the MP is more conserved, flagged by highly divergent N and C terminal regions. 

## 3. Discussion

Fruit tree viruses can cause infections with severe symptoms in many plant species thanks to various strategies they have evolved to overcome host defenses including RNA silencing [8]. One of the best studied ways they use to combat silencing is to encode one or more RSSs from their genomes. These proteins often differ greatly in their amino acid sequences, protein size, and mode of action [7]. Using a co-agroinfiltration procedure, we investigated whether the MP, CPL, and CPS proteins encoded by RNA2 of CVF are suppressors of RNA silencing. Out of the three proteins studied in the present work, only the MP exhibited some putative evidence of RSS activity. 

Previous work on broad bean wilt virus 2 (BBWV-2), another fabavirus member, has shown that VP53, VP37, and LCP proteins (encoded from RNA2 and involved with virus cell-to-cell and systemic movement) can suppress local RNA silencing triggered by ss GFP RNA [7]. These proteins were found to be able to reduce, but not to eliminate, siRNA accumulation locally interfering with the initial stages of RNA silencing [7]. In our study, none of the CVF CPL or CPS proteins were able to suppress RNA silencing, whereas for MP protein, a slight delay in the drop of fluorescence was observed in a number of replicates (11/92). These observations were further confirmed by the RT-qPCR results for the relative expression of GFP RNA. More specifically, a significant difference was recorded at 4 dpi between the GFP RNA levels of the empty vector control and CVF-MP (Figure 3). The variable intensity of GFP fluorescence observed at 6 dpi was also present in the expression levels of the three replicates, which had a mean similar to 4 dpi but with high SD (±1.39). Moreover, even though CVF-MP could not inhibit the cell-to-cell spread of the silencing signal, it interfered with systemic silencing in about 39% of the 16c plants triggered with GFP ssRNA and in approximately 68% of the plants induced with GFP ds RNA. Overall, our results indicate that CVF MP protein is implicated in the suppression of RNA silencing. More research is needed to elucidate the potential interference of MP with the RNA silencing pathway, also putatively utilizing different approaches e.g., expressed from a heterologous virus-based expression vector [9]. 

Sequence comparisons of the proteins under study were performed for the identification of the presence of conserved tryptophan–glycine and/or glycine–tryptophan (GW/WG)-rich motifs. These are AGO (Argonaute)-binding domains which bind AGO proteins that are essential during RNAi-mediated gene silencing [10]. From our analysis, no WG/GW-rich regions were identified in the proteins, suggesting that they are putatively not capable of interaction with AGOs in a GW/WG manner. Comparative sequence analysis of the CVF MP from different virus isolates showed rather high amino acid sequence divergence, indicating that there might be differences in the strength of the activity of the MP protein among the CVF isolates. Interestingly, the amino acid conservation was higher for the central part of the protein, suggesting that it could be implicated in its activity as a suppressor of the RNA silencing mechanism. The same intra-species diversity of CVF MP was observed in prunus virus F (PrVF), the closest fabavirus member [11].

## 4. Materials and Methods

### 4.1. Plant Material

Wild type and the GFP-expressing 16c line (16c) *Nicotiana benthamiana* plants were used for the transient expression experiments. All plants were reared in a growth chamber at 24 °C with 16 h light and 8 h darkness per day.

### 4.2. RNA Extraction

For the construction of expression clones, total RNA (T-RNA) was purified from freeze-dried tissue according to the protocol by White et al. [12]. Isolate SwC-G15-3 (LT991640) [4] was used.

### 4.3. Construction of Expression Clones

CVF’s mature peptides were determined using the alignment of the RNA2 polyproteins of all fabaviruses, which was constructed with MAFFT [13]. The cleavage sites were predicted by comparing CVF’s RNA2 polyprotein with known mature peptides of fabaviruses. The mature peptides predicted for CVF’s G15-3 isolate correspond to RNA2’s polyprotein aminoacids 1–345 for MP, 346–718 for CPL, and 719–869 for CPS. The primers were designed on these regions and in the cases of the forward primers for CPL and CPS and the reverse for MP and CPL, an ATG initiation codon and a TAA stop codon were added, respectively (Supplementary Table S1). 

Reverse transcription of the viral RNA was performed using Primescript RTase (Takara Bio, Inc.), according to the enzyme’s specifications, using each protein’s reverse primer as the RT primer. The mixture was incubated at 42 °C for 60 min followed by 70 °C for 15 min. For the amplification of each target sequence, a touchdown PCR was performed in a 25 μL final volume reaction using 0.5 units of Q5 high-fidelity DNA polymerase (NEB, Inc., Ipswich, MA, USA), 5 μL of 5x Q5 reaction buffer, 0.2 mM of each dNTP, 0.5 μM of each primer, 2 μL of the RT reaction as template, and molecular-biology-grade water up to the final volume. The mixture was incubated in a Labcycler Gradient (SensoQuest, GmbH, Göttingen, Germany) thermocycler using the following protocol. Samples were incubated at 98 °C for 30 s followed by 7 cycles segmented into 98 °C for 10 s, 55 °C with an increment of −0.8 °C/cycle for 20 s, and 72 °C for 45 s, followed by 40 cycles segmented into 98 °C for 10 s, 63 °C for 20 s, and 72 °C for 45 s, with a final extension step of 72 °C for 5 min. The amplicons were analyzed in a 1.2% agarose gel and visualized under UV light. The bands were excised with a scalpel and purified using a Monarch^®^ DNA Gel Extraction Kit (NEB, Inc.). 

pART7 and pART27 [14] plasmids were purified from *Escherichia coli* cultures using a Monarch^®^ Plasmid Miniprep Kit (NEB, Inc.). Digestions were carried out using the appropriate restriction endonucleases (NEB, Inc.) according to the manufacturer’s specifications. Dephosphorylation [P-] of the vectors was performed using antarctic phosphatase (NEB, Inc.) according to the manufacturer’s specifications. Ligations were performed using T4 DNA ligase (NEB, Inc.) according to the manufacturer’s specifications, with a 1:3 vector:insert molar ratio. For the transformation of pART7 and pART27 constructs, Invitrogen’s subcloning efficiency DH5α competent cells (ThermoFisher Scientific, Waltham, MA, USA) were used and transformation was carried out according to the manufacturer’s specifications. *Agrobacterium tumefaciens* clones harboring the constructs were acquired by transforming competent C58C1 cells (kindly provided by Prof. Kriton Kalantidis) using the freeze–thaw method [15].

### 4.4. Agroinfiltrations and GFP Imaging

Ten ml of LB broth (10 g peptone, 10 g NaCl, and 5 g yeast extract per liter) containing the appropriate antibiotics was inoculated with a single colony of an *A. tumefaciens* clone and incubated at 28 °C for two days with constant shaking (~230 rpm). After two days, 500 μL of the culture was inoculated in ten ml LB containing the appropriate antibiotics, 10 mM MES (pH 5.7), and 25 μΜ acetosyringone, and were grown overnight at 28 °C with constant shaking (~230 rpm). The overnight cultures were centrifuged at 2000× *g* for 10 min, the supernatant was decanted, and the pellet was washed with 5 mL infiltration buffer [10 mM MES (pH 5.7), 10 mM MgCl2, 150 μΜ acetosyringone] and centrifuged at 2000× *g* for 10 min. The supernatant was decanted, and the pellet was reconstituted in infiltration buffer for a final OD600 between 0.45 and 0.55. The cultures were kept in the dark for a minimum of 3 h before infiltrating the abaxial surface of the *N. benthamiana* leaf. A spot of approximately 2 cm in diameter was infiltrated for each construct. 

To investigate the capacity of CVF proteins to suppress silencing, we infiltrated WT *N. benthamiana* plants with the constructs indicated in Table 1. The different *A. tumefaciens* clones were mixed after incubation in the dark in a 1:1 ratio, just prior to the infiltrations. A clone expressing the p19 protein of cymbidium ringspot virus (At:35S:p19) was used as a positive control and one harboring a plasmid with the empty pART27 vector as the negative (At:35S:pART27); a GFP-expressing clone (pBIN-mGFP4, At:35S:GFP) [16] and a clone expressing an inverted repeat sequence of GFP (At:35S:GFP-hp) [17] were used for the induction of silencing (all clones were kindly provided by Prof. Kriton Kalantidis). To investigate the suppression of cell-to-cell and systemic silencing for CVF-MP, 16c plants were infiltrated with the constructs indicated in Table 1. After the infiltration, the plants were grown in a chamber at 24 °C with 16 h light and 8 h darkness per day. The plants were monitored for at least 10 days post infiltration to observe the reduction in GFP fluorescence for local silencing suppression and for 40 days to assess the interference with systemic silencing. A UVP high-intensity UV lamp was used for GFP excitation and plants were photographed using a digital camera.

### 4.5. Relative Expression of GFP

Wild type *N. benthamiana* plants were infiltrated with At:35S:pART27/At:35S:GFP, At:35S:CVF-MP/At:35S:GFP, and At:35S:p19/At:35S:GFP (as described in Section 4.4). Four, six, and eight days post infiltration, leaf discs were excised using a core borer with a ~1.7 cm diameter from the infiltrated spot. The disks were flash frozen in liquid N_2_ and were stored at −80 °C until they were used for total RNA extraction according to the protocol by Ruiz-García et al. [18] with modifications by Panailidou et al. [19] (including the on-column DNaseI treatment). Three leaves were used as biological replicates per timepoint. cDNA was synthesized with SuperScript II reverse transcriptase (Invitrogen, Waltham, MA, USA), with 18-mer oligo(dT) primer and 38.4 ng of total RNA, according to the manufacturer’s specifications. qPCR was performed using Xpert Fast SYBR 2X mastermix (GRiSP, Lda., Porto, Portugal) with ROX according to the manufacturer’s specifications with the addition of 2 μL of the RT reaction and 0.4 μΜ of each primer. Primer pairs GFP-ER Taq-F/GFP-ER Taq-R [20] and L23-F/L23-R [21] were used for the detection of GFP and L23 internal control, respectively. All qPCRs were performed in a StepOnePlus real-time PCR system (Applied Biosystems, Waltham, MA, USA) using three technical replicates for each sample in the following thermocycling protocol: initial denaturation at 95 °C for 5 min followed by 40 cycles segmented at 95 °C for 10 s and 60 °C for 20 s with a final melting curve stage of 95 °C for 15 s, 60 °C for 1 min, and a +0.3 °C ramp up to 95 °C for 15 s. Relative expression levels of GFP mRNA were calculated according to the 2^−ΔΔCq^ method. 

### 4.6. Sequence Analysis of the Tested Proteins

To identify whether the proteins under study possess Argonaute (Ago) binding capacity as a silencing suppression mechanism, all three protein sequences (MP, CPL, CPS) were tested using the Agos bioinformatic method [10] for the presence of conserved WG/GW-rich domains.

In addition, the CVF MP sequence (Isolate SwC-G15-3, accession number LT991640) was aligned using the MUSCLE algorithm available in MEGA 7 against homologous sequences available in the GenBank database, to evaluate the level of sequence divergence and track putative conserved domains.

## Figures and Tables

**Table 1:** Combinations of clones used in agroinfiltration for the evaluation of RNA silencing suppressing activity.

Silencing Mode	Plant Line Used	Clones Combination	No. of Replications
Local	WT	At:35S:CVF-MP/At:35S:GFP At:35S:CVF-CPL/At:35S:GFP, At:35S:CVF-CPS/At:35S:GFP At:35S:p19/At:35S:GFP, At:35S:pART27/At:35S:GFP	42
Local	WT	At:35S:CVF-MP/At:35S:GFP At:35S:p19/At:35S:GFP, At:35S:pART27/At:35S:GFP	50
Cell-to-cell	16c	At:35S:CVF-MP/At:35S:GFP At:35S:p19/At:35S:GFP At:35S:pART27/At:35S:GFP	20
Systemic, induced by GFP ssRNA	16c	At:35S:CVF-MP/At:35S:GFP At:35S:p19/At:35S:GFP At:35S:pART27/At:35S:GFP	18
			5
			5
Systemic, induced by GFP dsRNA	16c	At:35S:CVF-MP/At:35S:GFP-hp At:35S:p19/At:35S:GFP-hp At:35S:pART27/At:35S:GFP-hp	19
			5
			5

## Data Availability

The datasets generated during and/or analyzed during the current study are available from the corresponding author on reasonable request.

## Supplementary Materials

The following supporting information can be downloaded at: https://www.mdpi.com/article/10.3390/plants13020264/s1, Figure S1: Agroinfiltrations of 16c *Nicotiana benthamiana* leaves with At:35S:CVF-MP/At:35S:GFP for assessing the inhibition of the long-distance silencing signal induced by ssRNA GFP; Figure S2: Agroinfiltrations of 16c *Nicotiana benthamiana* leaves with At:35S:p19/At:35S:GFP-hp (A), At:35S:pART27/At:35S:GFP-hp (B), and At:35S:CVF-MP/At:35S:GFP-hp (C) for assessing the inhibition of the long-distance silencing signal induced by dsRNA GFP; Figure S3: Alignment of the RNA2 polyprotein amino acid (aa) sequences from different CVF isolates used in this study; Table S1: Primers used for the amplification of genes of interest from the genome of cherry virus F.

## References

[1] Kalantidis K., Schumacher H.T., Alexiadis T., Helm J.M. (2008). RNA silencing movement in plants. Mol. Biol. Cell.

[2] Silhavy D., Burgyan J. (2004). Effects and side-effects of viral RNA silencing suppressors on short RNAs. Trends Plant Sci..

[3] Levy A., Dafny-Yelin M., Tzfira T. (2008). Attacking the defenders: Plant viruses fight back. Trends Microbiol..

[4] Koloniuk I., Sarkisova T., Petrzik K., Lenz O., Přibylová J., Fránová J., Spak J., Lotos L., Beta C., Katsiani A. (2018). Variability studies of two Prunus-infecting fabaviruses with the aid of high-throughput sequencing. Viruses.

[5] Jo Y., Choi H., Cho J.K., Cho W.K. (2021). First Report of Cherry Virus F Infecting Japanese Plum in Korea. Plant Dis..

[6] Lee E., Vansia R., Phelan J., Lofano A., Smith A., Wang A., Bilodeau G.J., Pernal S.F., Guarna M.M., Rott M. (2023). Area Wide Monitoring of Plant and Honey Bee (*Apis mellifera*) Viruses in Blueberry (*Vaccinium corymbosum*) Agroecosystems Facilitated by Honey Bee Pollination. Viruses.

[7] Kong L.F., Wang Y.Q., Yang X.L., Sunter G., Zhou X.P. (2014). Broad bean wilt virus 2 encoded VP53, VP37 and large capsid protein orchestrate suppression of RNA silencing in plant. Virus Res..

[8] Burgyan J., Havelda Z. (2011). Viral suppressors of RNA silencing. Trends Plant Sci..

[9] Fan L., He C., Gao D., Xu T., Xing F., Yan J., Zhan B., Li S., Wang H. (2022). Identification of Silencing Suppressor Protein Encoded by Strawberry Mottle Virus. Front. Plant Sci..

[10] Zielezinski A., Karlowski W.M. (2011). Agos—A universal web tool for GW Argonaute-binding domain prediction. Bioinformatics.

[11] Villamor D.E., Pillai S.S., Eastwell K.C. (2017). High throughput sequencing reveals a novel fabavirus infecting sweet cherry. Arch. Virol..

[12] White E.J., Venter M., Hiten N.F., Burger J.T. (2008). Modified Cetyltrimethylammonium bromide method improves robustness and versatility: The benchmark for plant RNA extraction. Biotechnol. J..

[13] Katoh K., Standley D.M. (2013). MAFFT multiple sequence alignment software version 7: Improvements in performance and usability. Mol. Biol. Evol..

[14] Gleave A.P. (1992). A versatile binary vector system with a T-DNA organisational structure conducive to efficient integration of cloned DNA into the plant genome. Plant Mol. Biol..

[15] Annamalai P., Rao A.L.N. (2006). Delivery and expression of functional viral RNA genomes in planta by agroinfiltration. Curr. Protoc. Microbiol..

[16] Hasseloff J., Siemering K.R., Prasher D.C., Hodge S. (1997). Removal of a cryptic intron and subcellular localization of green fluorescent protein are required to mark transgenic Arabidopsis plants brightly. Proc. Natl. Acad. Sci. USA.

[17] Kościańska E., Kalantidis K., Wypijewski K., Sadowski J., Tabler M. (2005). Analysis of RNA silencing in agroinfiltrated leaves of Nicotiana benthamiana and Nicotiana tabacum. Plant Mol. Biol..

[18] Ruiz-García A.B., Bester R., Olmos A., Maree H.J. (2019). Bioinformatic tools and genome analysis of Citrus tristeza virus. Citrus Tristeza Virus.

[19] Panailidou P., Galeou A., Beris D., Pappi P., Theologidis I., Tzagaki E., Lotos L., Varveri C., Katis N.I., Maliogka V.I. (2023). Identification and genetic diversity of grapevine virus L in Greece. Arch. Virol..

[20] Costa Â., Marques N., Nolasco G. (2014). Citrus tristeza virus p23 may suppress systemic silencing but is not related to the kind of viral syndrome. Physiol. Mol. Plant Pathol..

[21] Liu D., Shi L., Han C., Yu J., Li D., Zhang Y. (2012). Validation of reference genes for gene expression studies in virus-infected Nicotiana benthamiana using quantitative real-time PCR. PLoS ONE.

