# Supplementary material for: Evaluation of the RNA Silencing Suppression Activity of Three Cherry Virus F-Encoded Proteins
Source: Plants (Basel). 2024 Jan 17;13(2):264. doi: 10.3390/plants13020264 (PMC10819124; doi:10.3390/plants13020264)
Supplement: Supplementary file 1 [file plants-13-00264-s001.zip › plants-2786091-supplementary.pdf]

Supplementary Table 1. Primers used for the amplification of genes of interest from the genome of cherry virus F.

**Primer Name**	**Sequence 5'-3'**	**Amplicon Size (nt)**
**MP-5'end**	TTTA**GAATTC**ATGTCTAGAACTGGTTACTCTG*	1057
**MP-3'end**	TTA**GGATCC**TTACTGTCCGATACTAG	
**LCP-5'end**	TTTA**GAATTC**ATGGCTATTGGATCATTAGAG	1144
**LCP-3'end**	TTA**GGATCC**TTACTGGGCACGAGAT	
**SCP-5'end**	TTTT**GAATTC**ATGTTTGAATCTTTTAGCAC	607
**SCP-3'end**	TTA**GGATCC**TTAGAGAGTGGCGATAC	

.

* Restriction endonuclease's recognition sites are highlighted in bold. ATG and UAA codons that are not present in cherry virus F genome are underlined.

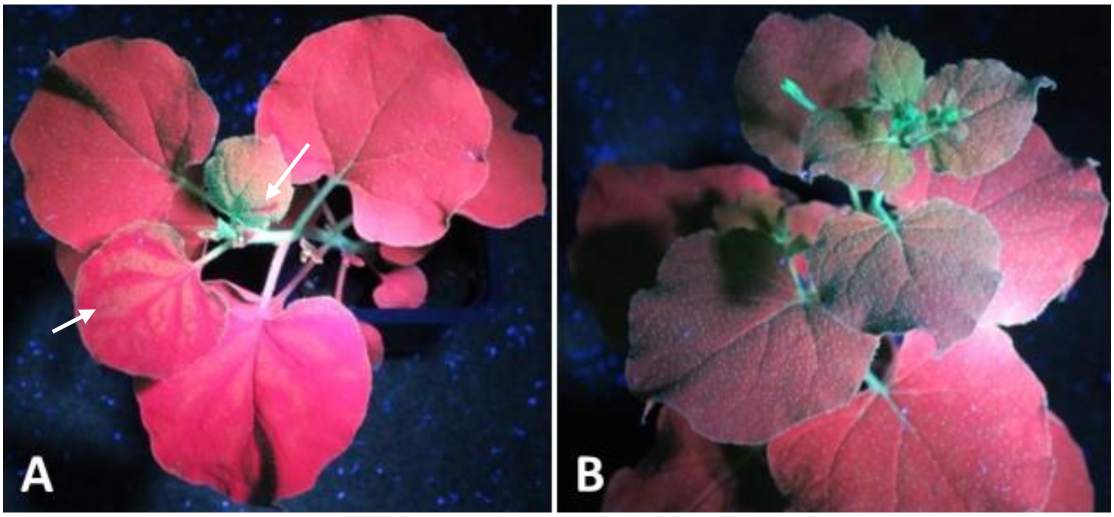

Supplementary Figure 1. Agroinfiltrations of 16c *Nicotiana benthamiana* leaves with At:35S:CVF-MP/At:35S:GFP for assessing the inhibition of the long-distance silencing signal induced by ssRNA GFP. Photos were taken at 24 dpi with plants infiltrated with cymbidium ringspot virus p19 protein retaining fluorescence whereas the negative control exhibited silenced nerves and leaves in the upper part of the plant (not shown here). Plants infiltrated with CVF-MP exhibited both phenotypes having either silenced leaves (A; white arrows) or leaves retaining fluorescence (B).

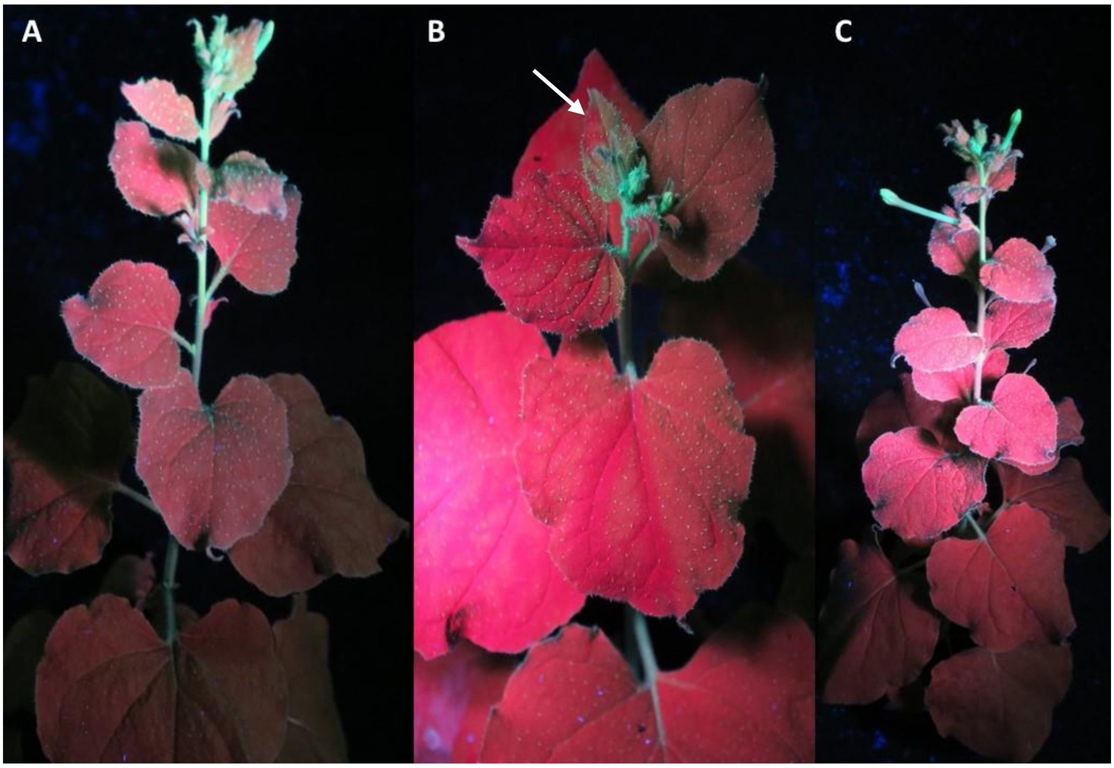

Supplementary Figure 2. Agroinfiltrations of 16c *Nicotiana benthamiana* leaves with At:35S:p19/At:35S:GFP-hp (A) At:35S:pART27/At:35S:GFP-hp (B) and At:35S:CVF-MP/At:35S:GFP-hp (C) for assessing the inhibition of the long-distance silencing signal induced by dsRNA GFP. Photos were taken at 24dpi (B) and 38dpi (A and C) with plants infiltrated with cymbidium ringspot virus p19 protein retaining fluorescence whereas the negative control exhibits silenced nerves and leaves in the upper part of the plant (white arrow). The majority of CVF-MP retained fluorescence after 40 days.

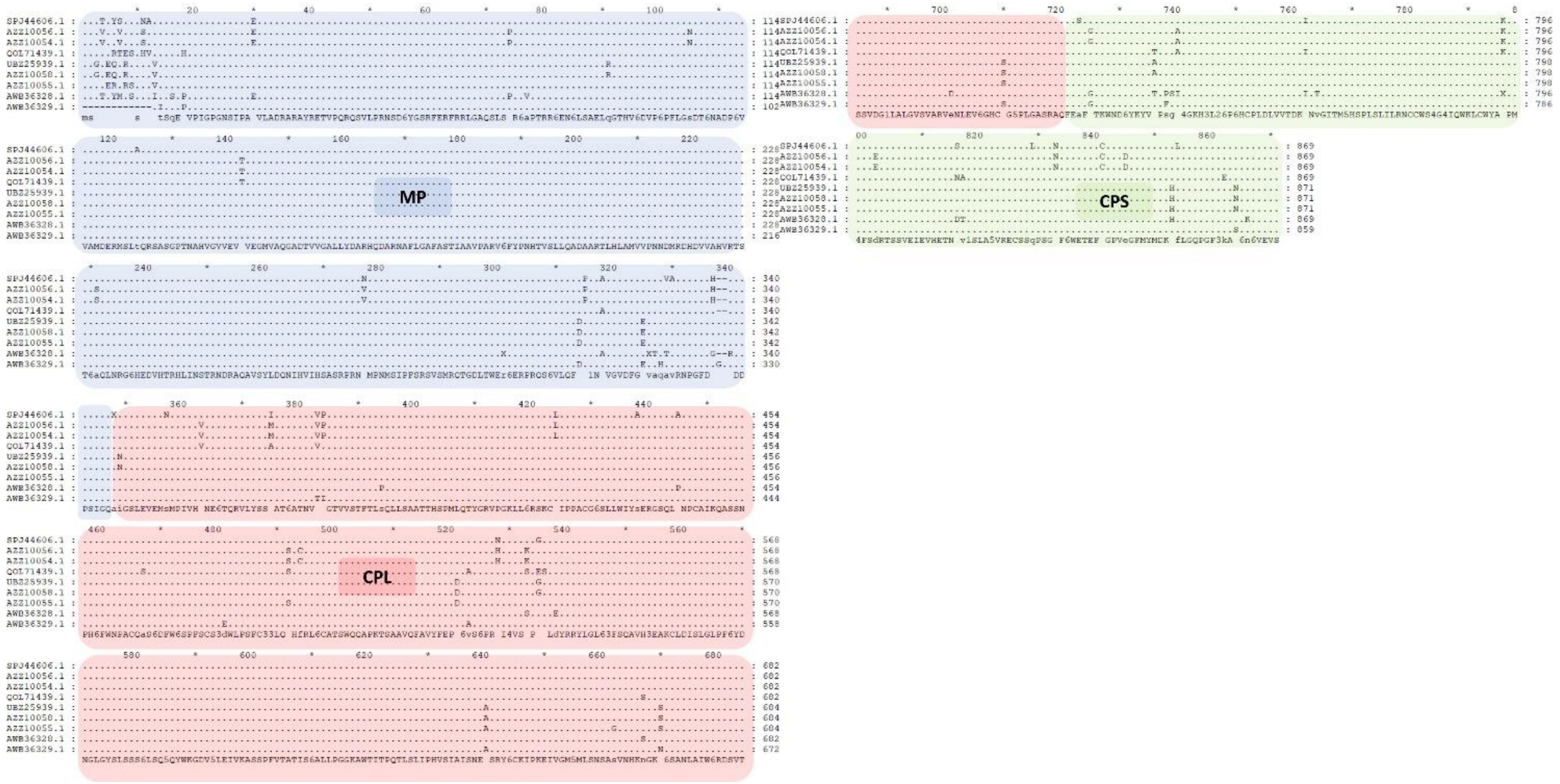

Supplementary Figure 3. Alignment of the RNA2 polyprotein amino acid (aa) sequences from different CVF isolates used in this study. MP, CPL and CPS protein sequences are indicated in blue, pink and green color, respectively. Available polyprotein sequences in GenBank of CVF isolates (SPJ44606, AWB36329, AZZ10056, AZZ10054, QOL71439, UBZ25939, AZZ10058, AZZ10055, AWB36328) were also included.
